# Application of functional quantum dot nanoparticles as fluorescence probes in cell labeling and tumor diagnostic imaging

**DOI:** 10.1186/s11671-015-0873-8

**Published:** 2015-04-10

**Authors:** Mei-Xia Zhao, Er-Zao Zeng

**Affiliations:** Key Laboratory of Natural Medicine and Immune Engineering, Henan University, Kaifeng, 475004 China

**Keywords:** Nanoparticles, Quantum dots, Optical properties, Biomaterials

## Abstract

Quantum dots (QDs) are a class of nanomaterials with good optical properties. Compared with organic dyes, QDs have unique photophysical properties: size-tunable light emission, improved signal brightness, resistance against photobleaching, and simultaneous excitation of multiple fluorescence colors. Possessing versatile surface chemistry and superior optical features, QDs are useful in a variety of *in vitro* and *in vivo* applications. When linked with targeting biomolecules, QDs can be used to target cell biomarkers because of high luminescence and stability. So QDs have the potential to become a novel class of fluorescent probes. This review outlines the basic properties of QDs, cell fluorescence labeling, and tumor diagnosis imaging and discusses the future directions of QD-focused bionanotechnology research in the life sciences.

## Review

Quantum dots (QDs) are a kind of semiconductor fluorescent semiconductors, which have gained attraction in recent years by scientists as a novel fluorescent probe [1-6]. Compared with conventional organic fluorescent probes, QDs have shown great potential to be fluorescent probes and images in biology because of their unique optical properties [7-15]. Alivisatos and Nie employed QDs as fluorescent probes in biological staining and diagnostics that provided a novel study to display QDs as labels for cell and tissue research [16,17]. In recent years, QDs have been widely used in many fields of life sciences as fluorescent markers [18-21]. Herein, we briefly review the basic properties of QDs and the application of functionalized QDs as fluorescent probes in biological systems.

### The unique properties of quantum dots

QDs have unique optical and electrical properties due to its quantum effect and size effect. When the size of a particle is of nanometer scale, it will cause quantum confinement effect, size effect, dielectric confinement effect, macroscopic quantum effect, and surface effect. Consequently, QDs exhibit many optical properties different from macroscopic materials, and they have a very broad application prospects in biological fluorescent probes and functional materials. Therefore, QDs will have a meaningful effect on the continued development of life sciences [22-28].

### Quantum dots have unique optical properties

**Figure 1:**
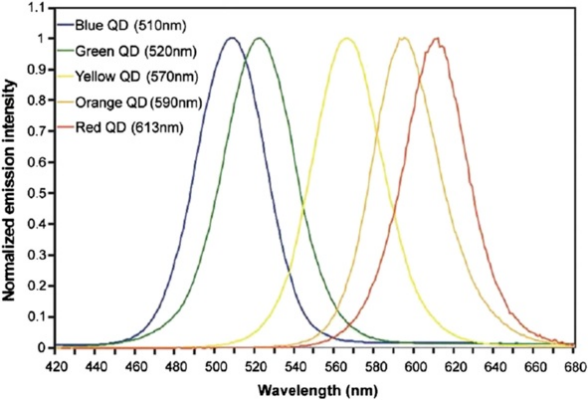
**Various QDs were excited at 350 nm and emission from 420 to 680 nm** **[** 32 **]** **.**

QDs have gained attraction owing to their unique fluorescence properties, which are due to the electron–hole and the interaction with the surrounding environment. When the excitation level of photon exceeds the band gap, the QDs will absorb photons to make the electrons transit from the valence band to the conduction band and shine. And if the composition and size of the QDs changed, they could obtain the emission spectra within the range from blue to red [29-31]. Conversely, when we want to obtain a variety of colors from multifarious fluorescent dyes, we often need a variety of excitation light, which not only increases the experimental costs but also makes the analysis system more complex. In addition, because of the optical properties of QDs, we can select more appropriate excitation wavelengths from their successive excitation spectra, which minimize the autofluorescence of biological samples to improve the resolution of the results (Figure 1) [32].

### Quantum dots have large stokes shift

**Figure 2:**
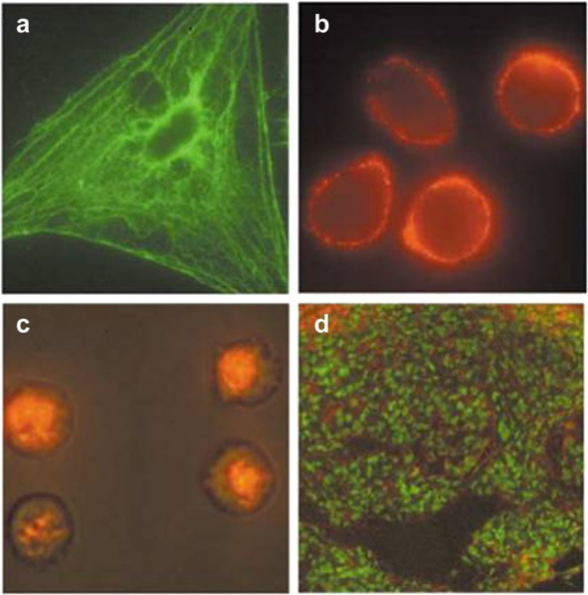
**Fluorescence micrographs of QD-stained cells and tissues. (a)** Actin staining on fixed 3 T3 fibroblast, **(b)** Live MDA-MB-231 breast and **(c)** mammalian cells. **(d)** Frozen tissue specimens and a nuclear dye [35].

Compared with the organic dyes, QDs have a larger stokes shift. Thereby, the spectroscopy detection can be achieved at a low signal intensity, which makes the fluorescence detection easier [7]. Biomedical organs usually have strong autofluorescence, and organic fluorescent dyes usually have a small stokes shift. A filter must be used when organic fluorescent dyes act as a fluorescence detector, which will greatly weaken the output intensity of fluorescence, and the detection signal will usually be covered by the autofluorescence of organs. Nevertheless, the fluorescence signal of QDs is able to overcome the impact of the autofluorescence and can be clearly distinguished from the background [33,34]. The fluorescence emission spectra of QDs are relatively narrow and can show different colors simultaneously without overlapping. So the fluorescence of different components can be marked simultaneously in experiments to obtain multicolor images (Figure 2) [35].

### Quantum dots have strong fluorescence intensity and high stability and strong resistance ability to photobleaching

**Figure 3:**
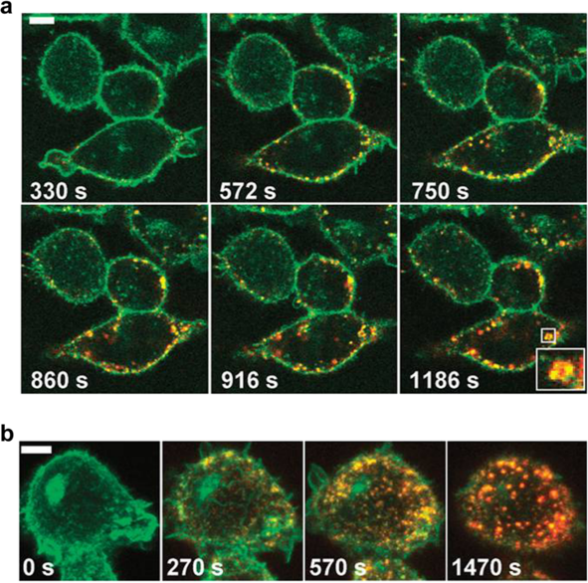
**QD probes enable long-term real-time monitoring of erbB1-eGFP receptor by EGF-QDs. (a)** CHO cells expressing erbB1-eGFP after addition of 200 pM 6:1 EGF-QDs. **(b)** CHO cells expressing erbB1-eGFP after addition of 250 pM 30:1 EGF-QDs. Scale bars 5 μm [37].

The fluorescence intensity of QDs is 20 times higher than the commonly used organic fluorescent dye Rhodamine 6G, and the stability of QDs is above 100 times than that of Rhodamine 6G, and at the same time, QDs have strong ability to resist photobleaching [35]. The photobleaching is a phenomenon that the luminescent material decomposes due to optical excitation, leading to the decrease of fluorescence intensity. However, the photobleaching rate of organic fluorescent dyes is very fast, and organic fluorophores will start irreversible light chemical reaction with light stimulates, resulting in a loss of fluorescence, yet the photobleaching rate of QDs is much lower. For example, the fluorescence intensity of modified CdSe/ZnS QDs hardly diminished at 500 mW, 488 nm excitation for 14 h [32]. Based on the high photochemical stability of resisting photobleaching, the cells and organs labeled by QDs can be observed for a long time (Figure 3) [36,37], unlike the traditional organic fluorescent dyes, which are prone to fluorescence quenching. This also provides a powerful tool for the study of long-term cellular interactions between biological molecules [38].

### Quantum dots have long fluorescence lifetime

The fluorescence lifetime of typical organic fluorescent dyes is only a few nanoseconds (ns), which is similar to the decay time of autofluorescence of biological samples. While the fluorescence lifetime of QDs can sustain tens of nanoseconds (20 to 50 ns), which makes the fluorescence of QDs still persist and obtain fluorescence signals without background interference when the majority of the auto-fluorescence background has been attenuated after a few nanoseconds of light excitation [39,40].

### Quantum dots have good biocompatibility

The chemically modified QDs are less harmful to organisms with good biocompatibility and low cytotoxicity. And it is easy to achieve QD surface functionalization of specific connection and biological labeling and detection *in vivo* after a variety of chemical modifications [41].

Since the surface chemical properties of the same kind of fluorescent nanoparticles of different sizes are very similar, the chemical modification method for particles of one size can also be applied to other sizes of particles, making it easy to obtain a series of fluorescent marking materials of the same surface-modified structure but of different optical properties, which greatly simplifies the biochemical modification process of fluorescent probes [42]. In addition, QDs are of good spatial compatibility, a QD can simultaneously couple two or more biological molecules or ligands, so QDs can be used to prepare multifunctional materials for detection and imaging [43,44]. Because of these unique optical properties, QDs can be used as ideal fluorescent probes [45]. And instead of organic fluorescent dyes, QDs will play an important role in the study in the cellular location, signal transduction, intracellular molecular movement, and migration [46,47].

### The applications of quantum dots in cell labeling and imaging

The most promising application of QDs is that they can be used as fluorescent probes in biological systems. QDs made of different materials coupled with biological molecules can replace a lot of fluorescent dye molecules and play a significant role in cell biology research. And the light stability of QD markers makes long-term tracking of biological molecules possible, using the labeling technique [48,49].

In the biomedical field, one of the earliest and most successful applications of QDs is in cell biology research; for example, QDs can be used as cell surface markers and intracellular markers [11]. Cell surface markers are obtained by specifically binding QDs with biomolecules to cell surface by streptavidin-biotin system, and the QDs are indirectly marked on cell surface. Based on the fact that green silanized QDs have the characteristics of high affinity with nuclei, Alivisatos et al. directly marked murine fibroblast cell nuclei using the QDs [50]. Taking advantage of electrostatic interactions, they connected avidin to the surface of red silanized QDs and labeled QDs to the F-actin on cell surface by the specificity of biotin-avidin. So, for the first time, the two-color QDs marked individual cells simultaneously.

**Figure 4:**
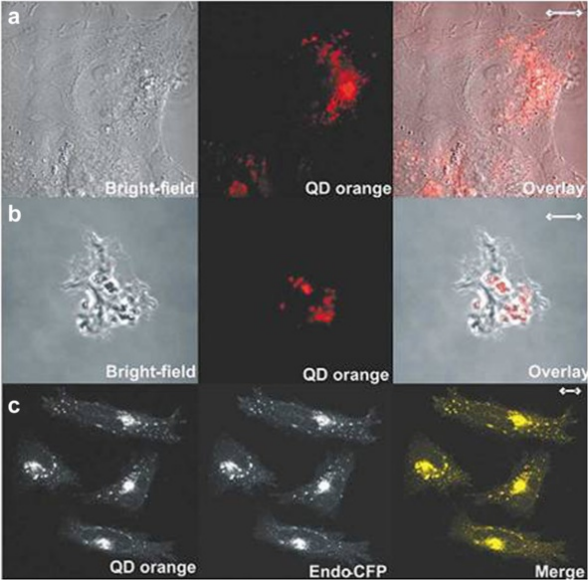
**Generalized labeling of living cells using QDs.** Confocal images of **(A)** HeLa and **(B)** D. discoideum cells. **(C)** Three-dimensional confocal projection of HeLa cells transiently transfected with the plasmid pECFP-Endo (encoding an endosome-specific reporter) and labeled with QD orange as above.[32].

In recent years, the application of QDs has been further widened in cell biology research, and the application in living cells is particularly noticeable [51,52]. Gerion et al. for the first time made the QDs and labeled molecule complexes enter the nuclei by transfection. In the experiment, QDs and T-antigen nuclear localization signal (NLS) of SV40 (simian virus 40) were combined and entered living cells after transfection. The motor process of the complexes from the cytoplasm to the nucleus was monitored by the fluorescence imaging system. After observation for more than a week, they found that QDs had no negative effect on the cells, and the accumulation of the complexes was in the nuclei [53]. In this work, QDs were for the first time used for real-time observation of biological phenomena in the nuclei for a long time, which offers a new non-cytotoxicity imaging technique to study the exchange mechanism and processes in nucleus. Meanwhile, Simon et al. also labeled living cells using CdSe/ZnS QDs, which did not affect the growth of normal cell and cell signaling (Figure 4) [32]. QDs mainly enter the cells by endocytosis, and the fluorescence performance of QDs in the cells can last over a week and does not affect cell morphology. Based on these progresses, we can achieve the purposes of long-term observation and the tracking of live cells marked by QDs of different colors.

**Figure 5:**
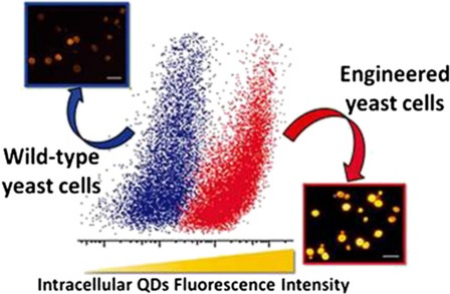
**The comparison of intracellular QDs fluorescence intensity of wild-type yeast cells and engineered yeast cells** **[** 54 **]** **.**

**Figure 6:**
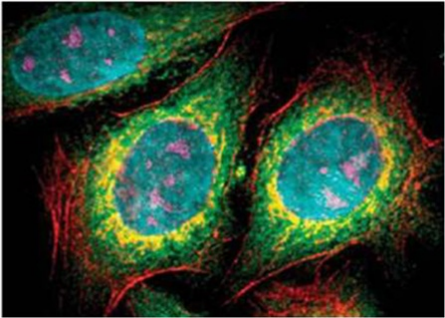
**The multicolor imaging of fixed human epithelial cells using five different color QDs** **[** 55 **]** **.**

**Figure 7:**
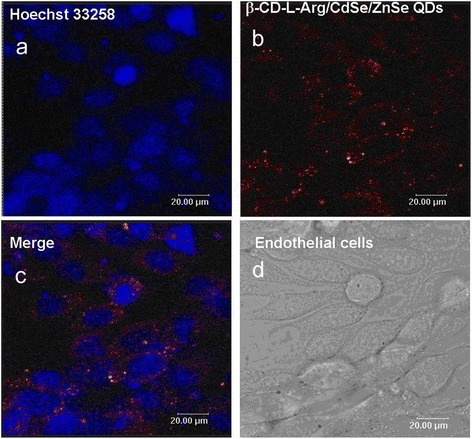
**Using confocal microscopy for imaging β-CD-L-Arg/CdSe/ZnSe QDs in ECV-304 cells. (a)** fluorescent images of Hoechst 33258-stained ECV-304 cells, **(b)** fluorescent images of ECV-304 cells with β-CD-L-Arg/CdSe/ZnSe QDs, **(c)** an overlay of bright field and fluorescent images of the β-CD-L-Arg/CdSe/ZnSe QDs labeled ECV-304 cells, **(d)** in the bright field images. [56]

**Figure 8:**
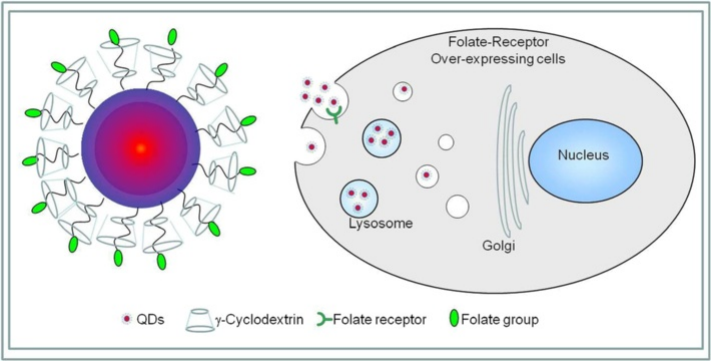
**The schematic of folate-receptor targeted γ-CD/FA-functionalized QDs using tumor-targeting FA in the presence of cell-penetrating β-CD** **[** 57 **]** **.**

Pang et al. have also made a number of breakthroughs in cell labeling and imaging research. For example, as an intracellular indicator, CdSe QD fluorescence was successfully used in genetic techniques, and they found that the glutathione metabolic pathway is important for the biosynthetic yield of CdSe QDs. And compared with the wild-type strain, the glutathione metabolic mutant strains showed significantly decreased fluorescence intensity and biosynthetic yield of CdSe QDs (Figure 5) [54]. Gao et al. realized five colors at a time with excitation by a single wavelength of light, namely, cyan QDs labeled nuclei; magenta QDs labeled Ki-67 protein; orange QDs marked mitochondria; green QDs labeled microtubules; and red QDs labeled actin (Figure 6) [55]. However, common markers and fluorescent dyes do not have the above features, so QDs have broad biological applications. Our group modified arginine β-cyclodextrin (β-CD) to the surface of QDs and got more evenly distributed QDs with smaller sizes, good solubility, good stability, and biocompatibility. Besides, the functionalized QDs have relatively good optical performance in aqueous solution. From Figure 7, we can see that the QDs can enter the cytoplasm of living ECV-304 cells to label the cell [56]. In addition, we synthesized folate receptor-targeted QDs with lower toxicity, good biocompatibility, good optical performance, and smaller sizes, using targeting folic acid (FA) molecule directly to bind the QDs surface. The experiment results of cellular localization with transmission electron microscopy and confocal fluorescence microscopy showed that the folate receptor-targeted QDs can effectively recognize folate receptor-overexpressed cancer cells through folate receptors and can be distributed within lysosomes (Figure 8) [57].

### The applications of quantum dots in tumor diagnostic

**Figure 9:**
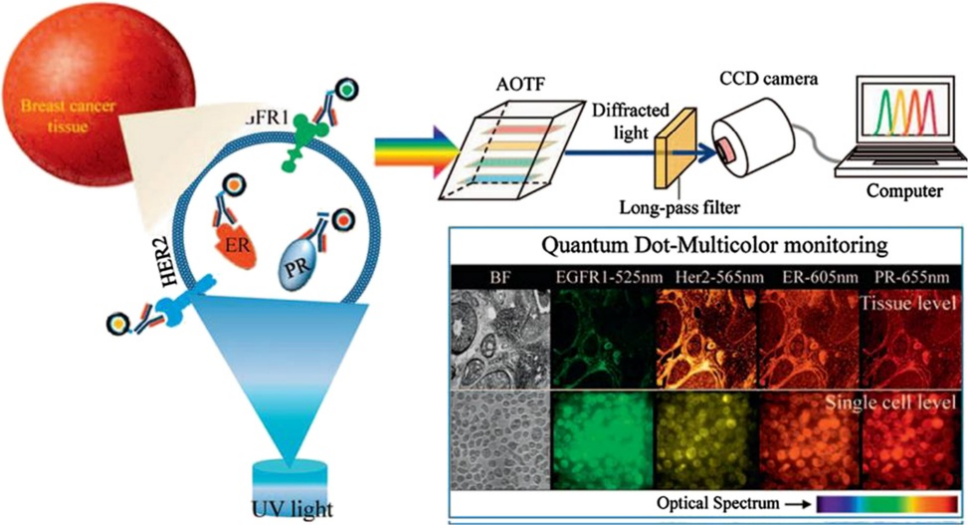
**A schematic representation of quantum dot based single cell imaging cytometry for the determination of breast cancer subtypes.** Biopsied tissues or primary cells from breast tumors were treated with four different quantum dot-biomarker (EGFR1, HER2, ER, and PR) conjugates and excited by UV light. Hyperspectral fluorescence cellular images were detected at 525 nm, 565 nm, 605 nm, and 655 nm with the help of acousto-optic tunable filter (AOTF). The optical spectrum represents increasing wavelength as a function of color i.e. blue to red. BF: bright field [59].

**Figure 10:**
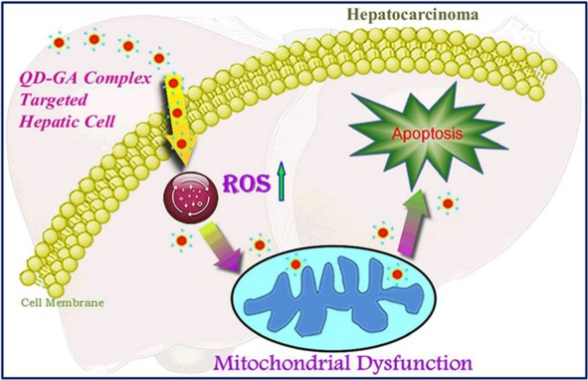
**QD-GA complex-targeted hepatic cells and induced apoptosis through an ROS-mediated mitochondrial dysfunction pathway** **[** 60 **]** **.**

The optical imaging technique based on fluorescent probes with QDs is a sensitive, non-ionizing, inexpensive technique, and it has great potential for early diagnosis of cancers [58]. Significant heterogeneous subtypes that have to be considered for proper drug treatment were determined depending on the tumor regions of breast cancer patients. Song et al. have successfully presented QD-based quantitative high-content imaging cytometry as a new model for breast cancer prognosis and classification [59]. In their experiments, four different receptors, epidermal growth factor receptor (EGFR1), HER2, estrogen receptor (ER), and progesterone receptor (PR), were conjugated to maleimide-functionalized quantum dots. And the four different QD-antibody conjugates (QD525-EGFR1, QD565-HER2, QD605-ER, and QD655-PR) were used as QD immunoassay probes for the subtype classification of biopsied samples from breast cancer patients (Figure 9). Moreover, our group synthesized β-CD/GA-functionalized QDs, which led to improved antitumor activity and induced apoptosis in hepatocarcinoma cells (Figure 10) [60].

Cervical cancer is one of the most fatal diseases, and researchers are actively developing methods of early diagnosis and treatment. Currently, QDs have been successfully applied to tumor imaging at the cellular level. Rahman et al. [61] combined overexpression SiHa cervical cancer cells of epidermal growth factor receptor (EGFR) with the monoclonal antibody of biotinylated EGFR and found that the fluorescence intensity of the experimental group was significantly higher than that of the control group after being irradiated by two kinds of excitation light of the confocal fluorescence microscopy. And the tumor cells could be identified from the full organization by the QD probes, which have a more competitive advantage than the traditional contrast agents. It proved that QD probes can detect cervical cancer at the molecular level, which provides a new way for early diagnosis of cervical cancer. The strong fluorescent stability of QDs also shows superiority in research on living animal. In recent years, Ren et al. have reported CdSeTeS QDs modified with alpha-thio-omega-carboxy poly(ethylene glycol) (HS-PEG-COOH), and the modified QDs were linked to anti-epidermal growth factor receptor (EGFR) antibodies [62]. QDs with the EGFR antibodies as labeling probes were successfully applied to targeted imaging for EGFR on the surface of SiHa cervical cancer cells through conjugation of QDs with the anti-EGFR antibodies. These preliminary results indicated that CdSeTeS QDs can become useful probes for *in vivo* targeted imaging and clinical diagnosis.

## Conclusions

With the development of nanotechnology and the improvement of QD marking technology, QDs are increasingly showing its great value and good prospects, especially in the optical and biological aspects, due to their unique physical and chemical properties. Certainly, far more than this, QDs are also a powerful tool for drug screening. QDs will become the most promising fluorescent markers; for example, QDs show great potential for cell imaging, fluorescence immunoassay, etc. Especially, QDs have made great progress in the cellular imaging in recent years, showing far more advantages than the traditional organic dyes. The technology of using QDs as fluorescent markers of living cells is gradually becoming mature, diverse, and practical. It can be predicted that the QD fluorescence technology will open up new horizons for researches in complicated life phenomena of living cells.

## References

[1] Yong K-T, Law W-C, Hu R, Ye L, Liu L, Swihart MT (2013). Nanotoxicity assessment of quantum dots: from cellular to primate studies. Chem Soc Rev..

[2] Park Y, Ryu YM, Jung Y, Wang T, Baek Y, Yoon Y (2014). Spraying quantum dot conjugates in the colon of live animals enabled rapid and multiplex cancer diagnosis using endoscopy. ACS Nano..

[3] Biju V (2014). Chemical modifications and bioconjugate reactions of nanomaterials for sensing, imaging, drug delivery and therapy. Chem Soc Rev..

[4] Taniguchi S, Green M, Rizvi SB, Seifalian A (2011). The one-pot synthesis of core/shell/shell CdTe/CdSe/ZnSe quantum dots in aqueous media for in vivo deep tissue imaging. J Mater Chem..

[5] Chakraborty A, Jana NR (2015). Design and synthesis of triphenylphosphonium functionalized nanoparticle probe for mitochondria targeting and imaging. J Phys Chem C..

[6] He X, Ma N (2014). An overview of recent advances in quantum dots for biomedical applications. Colloids Surf B: Biointerfaces..

[7] Wu P, Yan X-P (2013). Doped quantum dots for chemo/biosensing and bioimaging. Chem Soc Rev..

[8] Esteve-Turrillas FA, Abad-Fuentes A (2013). Applications of quantum dots as probes in immunosensing of small-sized analytes. Biosens Bioelectron..

[9] Viswanath A, Shen Y, Green AN, Tan R, Greytak AB, Benicewicz BC (2014). Copolymerization and synthesis of multiply binding histamine ligands for the robust functionalization of quantum dots. Macromolecules..

[10] Sekhosana KE, Antunes E, Khene S, D’Souza S, Nyokong T (2013). Fluorescence behavior of glutathione capped CdTe@ZnS quantum dots chemically coordinated to zinc octacarboxy phthalocyanines. J Lumin..

[11] Mattoussi H, Palui G, Na HB (2012). Luminescent quantum dots as platforms for probing in vitro and in vivo biological processes. Adv Drug Deliver Rev..

[12] Bai M, Bornhop DJ (2012). Recent advances in receptor-targeted fluorescent probes for in vivo cancer imaging. Curr Med Chem..

[13] Molaei M (2015). Synthesizing and investigating photoluminescence properties of CdTe and CdTe@CdS core-shell quantum dots (QDs): a new and simple microwave activated approach for growth of CdS shell around CdTe core. Electron Mater Lett..

[14] Aubert T, Soenen SJ, Wassmuth D, Cirillo M, Van Deun R, Braeckmans K (2014). Bright and stable CdSe/CdS@SiO2 nanoparticles suitable for long-term cell labeling. ACS Appl Mater Interfaces..

[15] Michalet X, Pinaud FF, Bentolila LA, Tsay JM, Doose S, Li JJ (2005). Diagnostics quantum dots for live cells, in vivo imaging, and diagnostics. Science..

[16] Bruchez M, Moronne M, Gin P, Weiss S, Alivisatos AP (1998). Semiconductor nanocrystals as fluorescent biological labels. Science..

[17] Chan WCW, Nie S (1998). Quantum dot bioconjugates for ultrasensitive nonisotopic detection. Science..

[18] Guyot-Sionnest P, Lhuillier E, Liu H (2012). A “mirage” study of CdSe colloidal quantum dot films, Urbach tail and surface states. J Chem Phys..

[19] Demchenko AP (2013). Beyond annexin V: fluorescence response of cellular membranes to apoptosis. Cytotechnology..

[20] Taylor A, Wilson KM, Murray P, Fernig DG, Lévy R (2012). Long-term tracking of cells using inorganic nanoparticles as contrast agents: are we there yet?. Chem Soc Rev..

[21] Shinchi H, Wakao M, Nagata N, Sakamoto M, Mochizuki E, Uematsu T (2014). Cadmium-free sugar-chain-immobilized fluorescent nanoparticles containing low-toxicity ZnS-AgInS2 cores for probing lectin and cells. Bioconjug Chem..

[22] Cassette E, Helle M, Bezdetnaya L, Marchal F, Dubertret B, Pons T (2013). Design of new quantum dot materials for deep tissue infrared imaging. Adv Drug Delivery Rev..

[23] Orte A, Alvarez-Pez JM, Ruedas-Rama MJ (2013). Fluorescence lifetime imaging microscopy for the detection of intracellular pH with quantum dot nanosensors. ACS Nano..

[24] Fang M, Yuan J-P, Peng C-W, Pang D-W, Li Y (2013). Quantum dots-based in situ molecular imaging of dynamic changes of collagen IV during cancer invasion. Biomaterials..

[25] Au GH (2012). Shih WY, Shih WH, Mejias L, Swami VK, Wasko K, et al. Assessing breast cancer margins ex vivo using aqueous quantum dot molecular probes. Int J Oncol..

[26] Biju V, Itoh T, Ishikawa M (2010). Delivering quantum dots to cells: bioconjugated quantum dots for targeted and nonspecific extracellular and intracellular imaging. Chem Soc Rev..

[27] Orr-Ewing AJ (2014). Perspective: bimolecular chemical reaction dynamics in liquids. J Chem Phys..

[28] Li XB, Li ZJ, Gao YJ, Meng QY, Yu S, Weiss RG (2014). Mechanistic insights into the interface-directed transformation of thiols into disulfides and molecular hydrogen by visible-light irradiation of quantum dots. Angew Chem Int Ed Engl..

[29] Chung H, Choi H, Kim D, Jeong S, Kim J (2015). Size-dependence of excitation energy-related surface trapping dynamics in PbS quantum dots. J Phys Chem C.

[30] Painuly D, Bhatt A, Krishnan VK (2013). Mercaptoethanol capped CdSe quantum dots and CdSe/ZnS core/shell: synthesis, characterization and cytotoxicity evaluation. J Biomed Nanotechnol..

[31] Gao XH, Cui YY, Levenson RM, Chung LW, Nie S (2004). In vivo cancer targeting and imaging with semiconductor quantum dots. Nat Biotechnol..

[32] Jaiswal JK, Mattoussi H, Mauro JM, Simon SM (2003). Long-term multiple color imaging of live cells using quantum dot bioconjugates. Nat Biotechnol..

[33] Zrazhevskiy P, Senaw M, Gao X (2010). Designing multifunctional quantum dots for bioimaging, detection, and drug delivery. Chem Soc Rev..

[34] Pilla V, Alves LP, Iwazaki AN, Andrade AA, Antunes A, Munin E (2013). Thermo-optical characterization of cadmium selenide/zinc sulfide (CdSe/ZnS) quantum dots embedded in biocompatible materials. Appl Spectrosc..

[35] Gao XH, Yarig L, Petros JA, Marshall FF, Simons JW, Nie S (2005). In vivo molecular and cellular imaging with quantum dots. Curt Opin Biotechnol..

[36] Zrazhevskiy P, Gao XH (2009). Multifunctional quantum dots for personalized medicine. Nano Today..

[37] Lidke DS, Nagy P, Heintzmann R, Arndt-Jovin DJ, Post JN, Grecco HE (2004). Quantum dot ligands provide new insights into erbB/HER receptor-mediated signal transduction. Nat Biotechnol..

[38] Liu H, Xu S, He Z, Deng A, Zhu J-J (2013). Supersandwich cytosensor for selective and ultrasensitive detection of cancer cells using aptamer-DNA concatamer-quantum dots probes. Anal Chem..

[39] Liu L, Miao Q, Liang G (2013). Quantum dots as multifunctional materials for tumor imaging and therapy. Materials..

[40] Wu XY, Liu HJ, Liu JQ, Haley KN, Treadway JA, Larson JP (2003). Immunofluorescent labeling of cancer marker Her2 and other cellular targets with semiconductor quantum dots. Nat Biotechnol..

[41] Tomczaka N, Jańczewski D, Hana M, Vancsoa GJ (2009). Designer polymer-quantum dot architectures. Prog Polym Sci..

[42] Saikia K, Deb P, Kalita E (2013). Sensitive fluorescence response of ZnSe(S) quantum dots: an efficient fluorescence probe. Phys Scr..

[43] Chi X, Huang D, Zhao Z, Zhou Z, Yin Z, Gao J (2012). Nanoprobes for in vitro diagnostics of cancer and infectious diseases. Biomaterials..

[44] Novio F, Simmchen J, Vázquez-Mera N, Amorín-Ferré L, Ruiz-Molina D (2013). Coordination polymer nanoparticles in medicine. Coord Chem Rev..

[45] Bilan R, Fleury F, Nabiev I, Sukhanova A. Quantum dot surface chemistry and functionalization for cell targeting and imaging. Bioconjug Chem. 2015, DOI:10.1021/acs.bioconjchem.5b00069. 10.1021/acs.bioconjchem.5b00069 25710410

[46] Zhong H, Zhang R, Zhang H, Zhang S (2012). Modular design of an ultrahigh-intensity nanoparticle probe for cancer cell imaging and rapid visual detection of nucleic acids. Chem Commun (Camb)..

[47] Sandvig K, Torgersen ML, Engedal N, Skotland T, Iversen TG (2010). Protein toxins from plants and bacteria: probes for intracellular transport and tools in medicine. FEBS Lett..

[48] Mansur AAP, Ramanery FP, Mansur HS (2013). Water-soluble quantum dot/carboxylic-poly (vinyl alcohol) conjugates: insights into the roles of nanointerfaces and defects toward enhancing photoluminescence behavior. Mater Chem Phys..

[49] Lyashchova A, Dmytruk A, Dmitruk I, Klimusheva G, Mirnaya T, Asaula V (2014). Optical absorption, induced bleaching, and photoluminescence of CdSe nanoplatelets grown in cadmium octanoate matrix. Nanoscale Res Lett..

[50] Alivisatos AP (1996). Semiconductor clusters, nanocrystals, and quantum dots. Science..

[51] Jie G, Zhao Y, Niu S (2013). Amplified electrochemiluminescence detection of cancer cells using a new bifunctional quantum dot as signal probe. Biosens Bioelectron..

[52] Liu BR, Winiarz JG, Moon J-S, Lo S-Y, Huang Y-W, Aronstam RS (2013). Synthesis, characterization and applications of carboxylated and polyethylene-glycolated bifunctionalized InP/ZnS quantum dots in cellular internalization mediated by cell-penetrating peptides. Colloids Surf B: Biointerfaces..

[53] Chen FQ, Gerion D (2004). Fluorescent CdSe/ZnS nanocrystal-peptide conjugates for long-term, nontoxic imaging and nuclear targeting in living cells. Nano Lett..

[54] Li Y, Cui R, Zhang P, Chen B-B, Tian Z-Q, Li L (2013). Mechanism-oriented controllability of intracellular quantum dots formation: the role of glutathione metabolic pathway. ACS Nano..

[55] Gao JH, Xu B (2009). Applications of nanomaterials inside cells. Nano Today..

[56] Zhao M-X, Xia Q, Feng X-D, Mao Z-W, Ji L-N, Wang K (2010). Synthesis, biocompatibility and cell labeling of L-arginine-functional β-cyclodextrin-modified quantum dot probes. Biomaterials..

[57] Zhao M-X, Huang H-F, Xia Q (2011). Ji L-N. Mao Z-W. γ-Cyclodextrin-folate complex-functionalized quantum dots for tumor-targeting and site-specific labeling. J Mater Chem..

[58] Lucky SS, Soo KC, Zhang Y (2015). Nanoparticles in photodynamic therapy. Chem Rev..

[59] Tak YK, Naoghare PK, Kim BJ, Kim MJ, Lee ES, Song JM (2012). High-content quantum dot-based subtype diagnosis and classification of breast cancer patients using hypermulticolor quantitative single cell imaging cytometry. Nano Today..

[60] Zhao M-X (2012). Ji L-N. Mao Z-W. β-Cyclodextrin/glycyrrhizic acid functionalised quantum dots selectively enter hepatic cells and induce apoptosis. Chem Eur J..

[61] Rahman M, Abd-El-Barr M, Mack V, Tkaczyk T, Sokolov K, Richards-Kortum R (2005). Optical imaging of cervical pre-cancers with structured illumination: an integrated approach. Gynecol Oncol..

[62] Yang F, Xu Z, Wang J, Zan F, Dong C, Ren J (2013). Microwave-assisted aqueous synthesis of new quaternary-alloyed CdSeTeS quantum dots; and their bioapplications in targeted imaging of cancer cells. Luminescence..

